# Anti-Platelet Properties of Phenolic Extracts from the Leaves and Twigs of *Elaeagnus rhamnoides* (L.) A. Nelson

**DOI:** 10.3390/molecules24193620

**Published:** 2019-10-08

**Authors:** Bartosz Skalski, Bogdan Kontek, Agata Rolnik, Beata Olas, Anna Stochmal, Jerzy Żuchowski

**Affiliations:** 1 Department of General Biochemistry, Faculty of Biology and Environmental Protection, University of Łódź, 90-236 Łódź, Poland; bartosz.skalski@biol.uni.lodz.pl (B.S.); bogdan.kontek@biol.uni.lodz.pl (B.K.); agata.rolnik@unilodz.eu (A.R.); 2 Department of Biochemistry, Institute of Soil Science and Plant Cultivation, State Research Institute, 24-100 Puławy, Poland; asf@iung.pulawy.pl (A.S.); jzuchowski@iung.pulawy.pl (J.Ż.)

**Keywords:** antiplatelet activity, adhesion, aggregation, *E. rhamnoides*, blood platelets

## Abstract

Sea buckthorn (*Elaeagnus rhamnoides* (L.) A. Nelson) is a small tree or bush. It belongs to the Elaeagnaceae family, and has been used for many years in traditional medicine in both Europe and Asia. However, there is no data on the effect of sea buckthorn leaves and twigs on the properties of blood platelets. The aim of the study was to analyze the biological activity of phenolic extracts from leaves and twigs of sea buckthorn in blood platelets in vitro. Two sets of extracts were used: (1) phenolic compounds from twigs and (2) phenolic compounds from leaves. Their biological effects on human blood platelets were studied by blood platelet adhesion, platelet aggregation, arachidonic acid metabolism and the generation of superoxide anion. Cytotoxicity was also evaluated against platelets. The action of extracts from sea buckthorn twigs and leaves was compared to activities of the phenolic extract (a commercial product from the berries of *Aronia melanocarpa* (Aronox^®^) with antioxidative and antiplatelet properties. This study is the first to demonstrate that extracts from sea buckthorn leaves and twigs are a source of bioactive compounds which may be used for the prophylaxis and treatment of cardiovascular pathologies associated with blood platelet hyperactivity. Both leaf and twig extracts were found to display anti-platelet activity in vitro. Moreover, the twig extract (rich in proanthocyanidins) displayed better anti-platelet potential than the leaf extract or aronia extract.

## 1. Introduction

Platelets are highly reactive cells activated through various specific membrane receptors by physiological agonists, such as adenosine diphosphate (ADP), thrombin and collagen, as well as non-physiological agonists. They also play an important role in hemostasis, this being the regulation of the flowing properties of blood. In the presence of agonists, blood platelets respond by adhering to various adhesive proteins, including collagen, forming platelet aggregates and secreting various compounds from granules. Moreover, various biochemical processes such as phosphoinositide hydrolysis, arachidonic metabolism and eicosanoid biosynthesis, and reactive oxygen species (ROS) generation, are involved in platelet activation [1,2]. However, uncontrolled platelet activation is also an important risk factor of cardiovascular diseases. For example, blood platelets may form pathogenic thrombi, which are responsible for acute ischemic events [2]. In developed countries, the greatest single cause of mortality is due to cardiovascular conditions, such as atherosclerosis and thrombosis; these are responsible for about 50% of all deaths each year in Europe [3,4,5]. 

The most widely-known and popular anti-platelet drug is acetylsalicylic acid (aspirin), which acts as a cyclooxygenase inhibitor, an enzyme involved in eicosanoid synthesis. The most common side effects of aspirin are indigestion, stomach aches and bleeding [1,2]. Hence, there is great interest in identifying new anti-platelet agents without side effects. Many experiments, both in vitro and in vivo, suggest that berries may contain substances that affect the functioning of blood platelets, including their high phenolic content [6]. Various berries, including aronia berries (*Aronia melanocarpa*), blueberries (*Vacconium myrtillus*) and grapes (*Vitis*) have been found to possess antioxidant and antiplatelet activities [6,7,8]. Studies indicate that the effects of these fruits on blood platelet activation are dependent on not only the concentration of berry phenolics or the class of phenolic compounds, but also the type of berry and the form of food products or medical preparations [6]. In addition, the consumption phenolic compounds present in fresh berries or berry products, such as berry extracts, have not been associated with any unwanted or toxic activity, including hematological or urinary effects [7,8,9,10,11,12,13,14,15,16,17]. 

Dietary supplements, including commercial products made from aronia berries (Aronox^®^) may inhibit platelet activation, by reducing platelet aggregation or eicosanoid synthesis [10]. Various studies have shown that sea buckthorn (*Elaeagnus rhamnoides* (L.) A. Nelson) berries and their products may have therapeutic and protective properties against cardiovascular diseases [11,12,13,14,15]. Sea buckthorn is a small tree or bush. It belongs to the Elaeagnaceae family, and it has been used for many years in traditional medicine in both Europe and Asia. Sea buckthorn fruits have been used for treating various diseases, including cardiovascular diseases, for many years and are described in Chinese medical literature [6,8]. The therapeutic potential of sea buckthorn oils against cardiovascular diseases has been associated with its high unsaturated fatty acid content [8]. In addition, the phenolic-rich fraction of sea buckthorn berries has also been found to demonstrate anti-platelet activity [16], and sea buckthorn leaves and twigs contain various bioactive compounds, including phenolic compounds, with antioxidant and anticoagulant properties [17]. However, the mechanism behind their influence on blood platelet activation remains unknown. Therefore, the aim of the present study was to determine the biological activity of extracts from the leaves and twigs of sea buckthorn against blood platelets in vitro. The following battery of standard tests was used to obtain a broad overview of the key mechanisms behind the beneficial action of phenolic compounds on cardiovascular diseases: blood platelet adhesion to collagen type I and fibrinogen, blood platelet aggregation induced by various physiological agonists, metabolism of thiol groups and glutathione (GSH) in tested blood cells, nonenzymatic lipid peroxidation in resting platelets, arachidonic acid metabolism (enzymatic lipid peroxidation) in platelets activated by thrombin, and platelet superoxide anion (O_2_^−^) production. In addition, the cellular safety of tested extracts was evaluated in vitro using a cytotoxicity test against human blood platelets, measuring extracellular lactate dehydrogenase (LDH) activity. The action of extracts from sea buckthorn twigs and leaves was compared to activities of the phenolic extract, a commercial extract from the berries of *Aronia melanocarpa* (Aronox^®^) with antioxidative and antiplatelet properties [18,19,20].

## 2. Results

**Figure 1:**
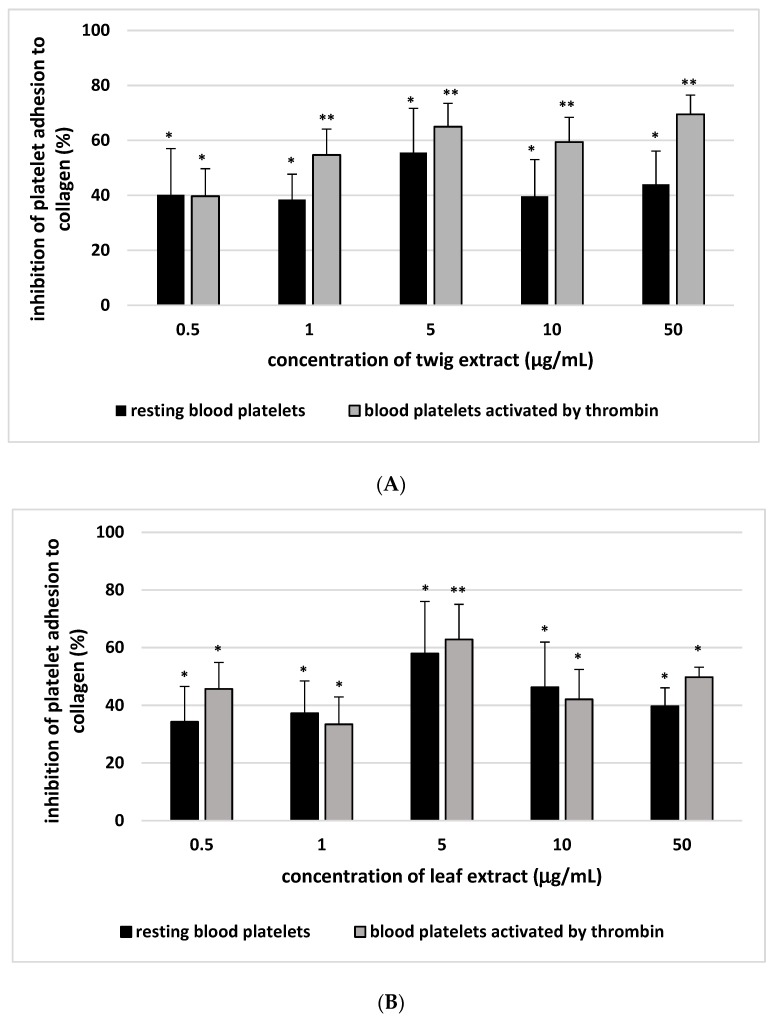
Twig (**A**) and leaf extract (**B**) (0.5–50 µg/mL; 30 min) on adhesion of resting blood platelets and thrombin-activated platelets to collagen. The inhibition of platelet adhesion by the plant extracts is expressed as the percentage of that recorded for control blood platelets (without the plant extract)–positive control. Data represent mean ± standard error (SE) of 5 (for resting platelets) and 9 (for thrombin-activated platelets) healthy volunteers (each experiment performed in triplicate). * *p* < 0.05, ** *p* < 0.02 (vs. control platelets).

**Figure 2:**
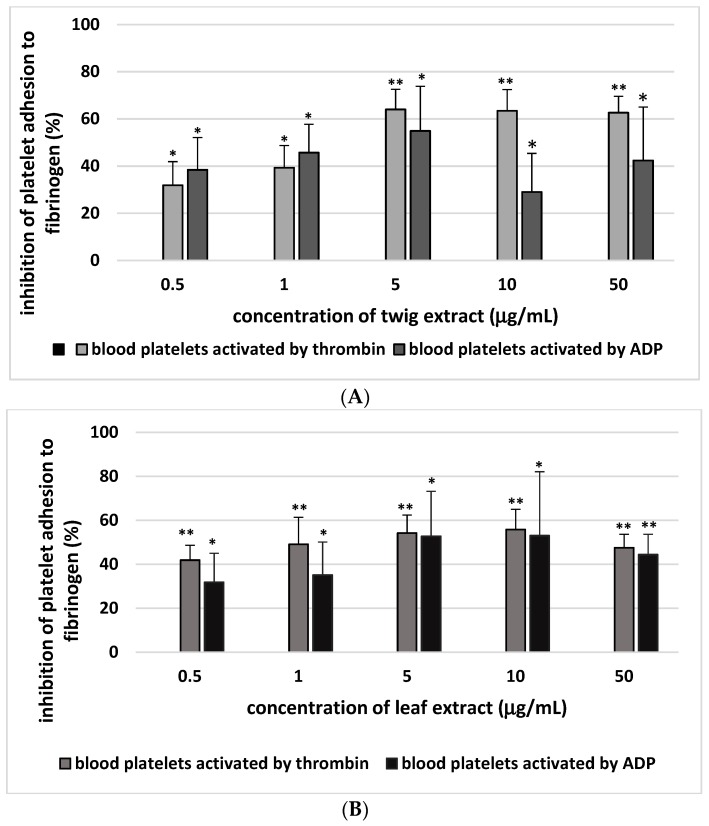
Twig (**A**) and leaf extract (**B**) (0.5–50 µg/mL; 30 min) on adhesion of thrombin/adenosine diphosphate (ADP)-activated platelets to fibrinogen. Inhibition of platelet adhesion by the plant extract is expressed as the percentage of that recorded for control blood platelets (without the plant extract)–positive control. Data represent mean ± SE of 5 (for ADP-activated platelets) and (for thrombin-activated platelets) healthy volunteers (each experiment performed in triplicate). * *p* < 0.05, ** *p* < 0.02 (vs. control platelets).

Our results show significantly lower adhesion to collagen of resting blood platelets and thrombin-activated platelets following preincubation with 0.5–50 µg/mL twig and leaf extract (Figure 1; Table 1). The percentage inhibition of adhesion of thrombin- or ADP-activated platelets to fibrinogen is given in Figure 2. At the highest tested concentration (50 µg/mL), the sea buckthorn twig extract demonstrated greater inhibition of thrombin-activated platelets to collagen or fibrinogen than the leaf extract. The twig extract demonstrated 69.5 ± 7.0% (*p* < 0.02) inhibition of adhesion to collagen and 62.6 ± 9.0% (*p* < 0.02) inhibition of adhesion to fibrinogen.

**Figure 3:**
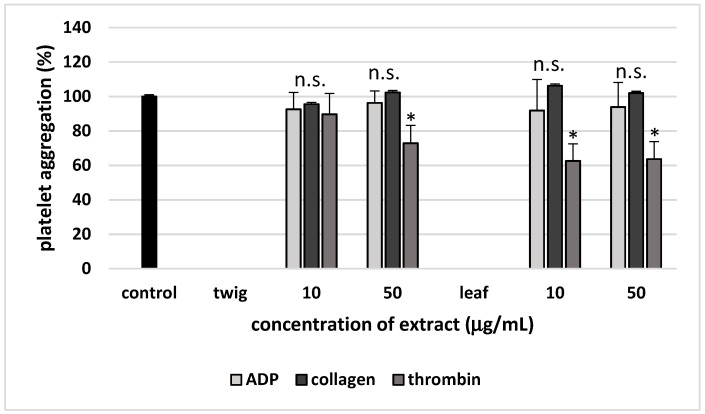
Effects of twig and leaf extract (10 and 50 µg/mL; 30 min) on blood platelet aggregation stimulated by different agonists: 10 µM ADP, 2 μg/mL collagen and 1 Unit/mL thrombin. Data represent mean ± SE of 5 (for thrombin-activated platelets) and 9 (for ADP or collagen-activated platelets) healthy volunteers (each experiment performed in triplicate). Neither concentration of the tested extract (10 and 50 µg/mL) had a statistically significant effect on aggregation stimulated by ADP and collagen compared to control platelets (*p* > 0.05 (n.s.)). However both concentrations of the tested extract (10 and 50 µg/mL) had a statistically significant effect on aggregation stimulated by thrombin compared to controls (* *p* < 0.05).

The next part of the study examined the potential of the twig and leaf extracts (at 10 and 50 µg/mL) to reduce platelet aggregation stimulated by different agonists, i.e., ADP, collagen and thrombin. The tested extracts were not found to display any anti-aggregatory properties when ADP and collagen were used as agonists (*p* > 0.05). However, both 10 and 50 µg/mL leaf extract inhibited thrombin-stimulated platelet aggregation, as did the twig extract at the higher concentration of 50 µg/mL (*p* < 0.05) (Figure 3). For example, the percentage inhibition of thrombin-stimulated platelet aggregation was 35.5 ± 9.1% (*p* < 0.05) for twig extract and 29.9 ± 8.9% (*p* < 0.05) for leaf extract at a concentration of 50 µg/mL (Figure 3).

**Figure 4:**
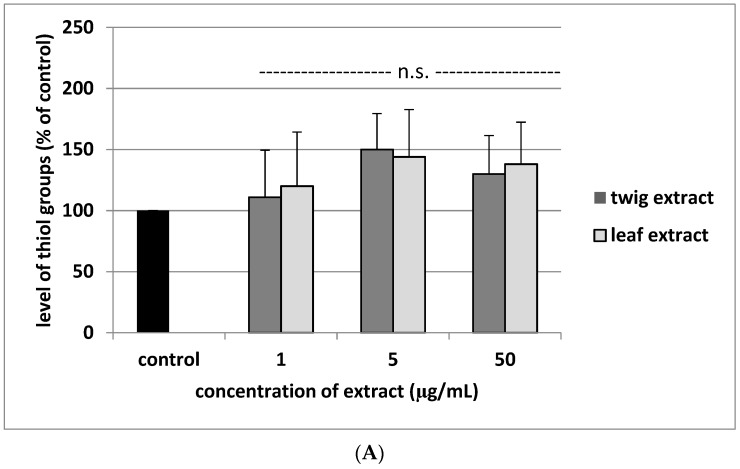

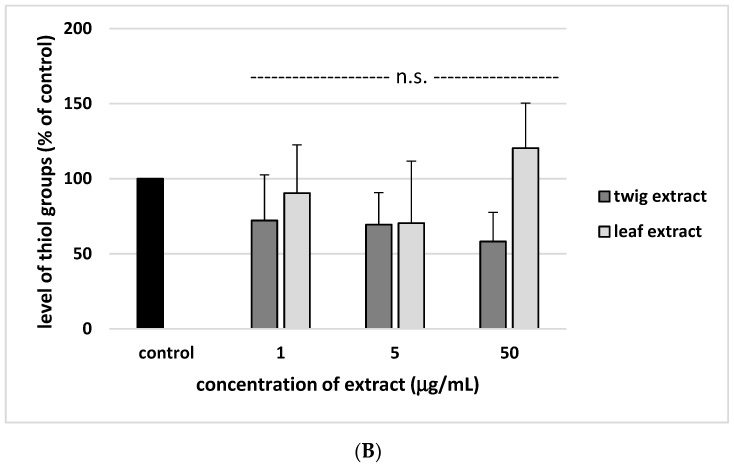
Twig and leaf extract (1, 5 and 50 µg/mL; 30 min) on the level of thiol groups in glutathione (GSH) fraction (**A**) and protein fraction (**B**) isolated from blood platelets. Data represent mean ± SE of 3 (for GSH) and 4 (for protein fraction) healthy volunteers (each experiment done in triplicate). In these experiments, the level of GSH fraction in control sample (positive control–blood platelets not treated with plant extract) was 5.7 ± 0.8 nmol GSH/mL of platelets, and was expressed as 100% (**A**); the level of thiol groups in protein fraction in control sample (positive control–blood platelets not treated with plant extract) was 112.4 ± 17.4 nmol GSH/mL of platelets, and was expressed as 100% (**B**). None of three different concentrations of the tested extract (1, 5 and 50 µg/mL) had a statistically significant effect compared to controls (*p* > 0.05 (n.s.)).

No change was observed in platelet GSH concentration or thiol group number in platelet proteins following exposure to the two tested sea buckthorn extracts at concentrations between 1–50 µg/mL (*p* > 0.05) (Figure 4).

**Figure 5:**
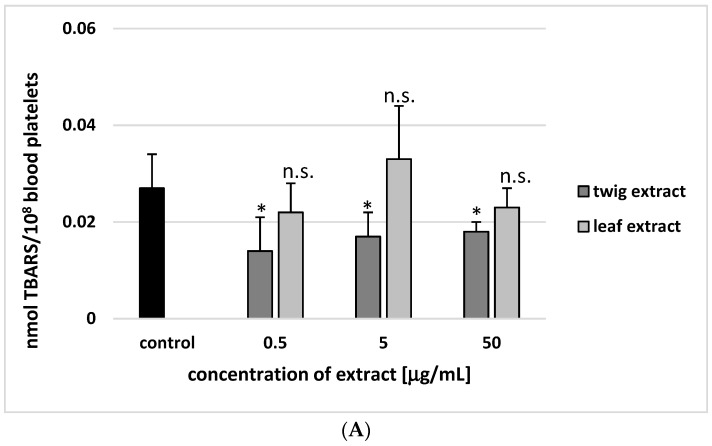

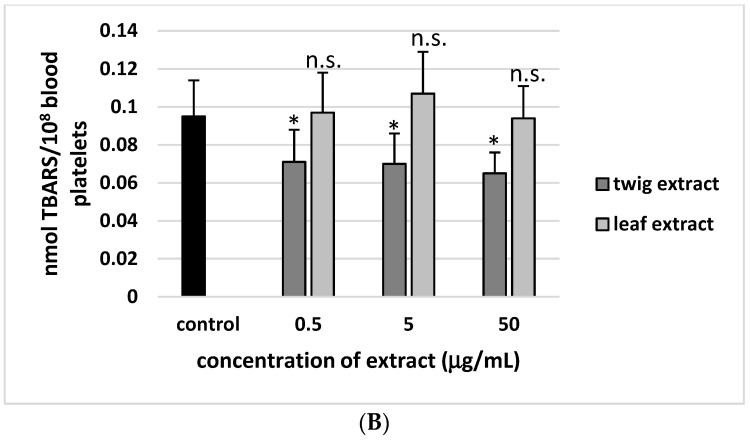
Twig and leaf extract (0.5, 5 and 50 µg/mL; 30 min) on lipid peroxidation in resting platelets (**A**) and in blood platelets activated by thrombin (**B**). In these experiments, blood platelets not treated with plant extract were used as control samples (positive control). Data represent mean ± SE of 6 healthy volunteers (each experiment done in triplicate). The three different concentrations of the twig extract (0.5, 5 and 50 µg/mL) had a statistically significant compared to controls (* *p* < 0.05). However, none of the three different concentrations of the leaf extract (0.5, 5 and 50 µg/mL) had any statistically significant effect compared to controls (*p* > 0.05 (n.s.)).

As demonstrated in Figure 5A,B, no change in the thiobarbituric acid reactive substances (TBARS) level was observed in the resting blood platelets or the thrombin-activated blood platelets following incubation with the leaf extract at concentrations of 0.5, 5 or 50 µg/mL (*p* > 0.05). On the other hand, all used concentrations of twig extract (0.5, 5 and 50 µg/mL) significantly reduced lipid peroxidation in both the resting and the thrombin-activated platelets (*p* < 0.05) (Figure 5A,B). At the highest-used concentration of twig extract (50 µg/mL), inhibition of lipid peroxidation was found to be about 40% for both resting platelets and those activated by thrombin (Figure 5A,B). In addition, 50 µg/mL twig extract demonstrated stronger inhibition than leaf extract at the same concentration.

**Figure 6:**
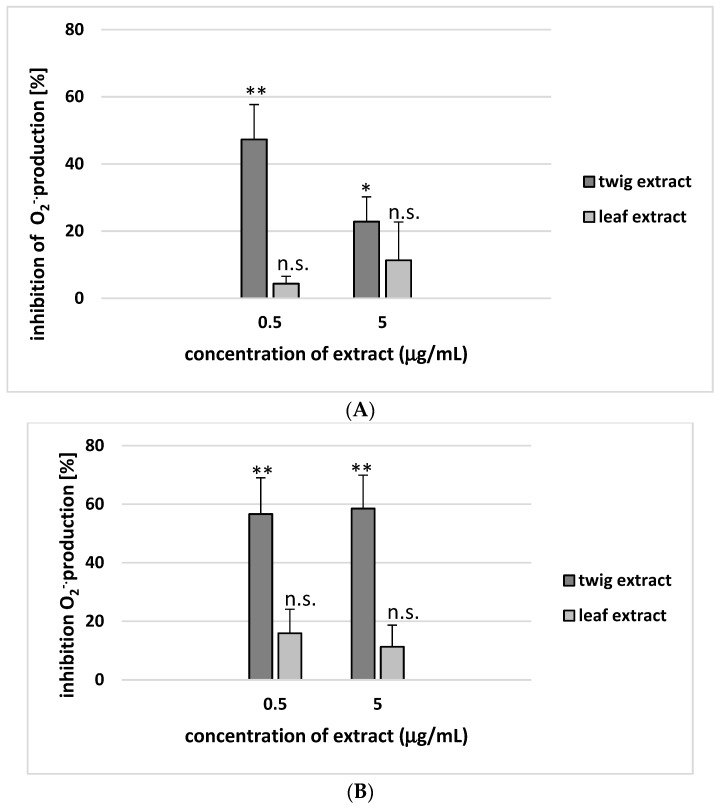
Effects of twig and leaf extract (0.5 and 5 µg/mL; 30 min) on O_2_^−^ production in resting platelets (**A**) and in blood platelets activated by thrombin (**B**). Data represent mean ± SE of 5 healthy volunteers (each experiment done in triplicate). In these experiments, the O_2_^−^ level in control samples (positive control – blood platelets not treated with plant extract) was 0.592 ± 0.321 nmol/10^8^ platelets (for resting platelets) and 1.222 ± 0.434 nmol/10^8^ platelets (for thrombin-activated platelets). Inhibition of O_2_^−^ production was expressed as a percentage of that recorded for positive control (platelets without tested extracts). The effects of the two different concentrations of twig extract (0.5 and 5 µg/mL) were significantly different to controls (* *p* < 0.05; ** *p* < 0.02). The two different concentrations of leaf extract (0.5 and 5 µg/mL) demonstrated no statistically significant effect compared to control platelets (*p* > 0.05 (n.s.)).

Only the twig extract was found to significantly reduce the process of O_2_^−^ production in resting platelets and activated platelets (Figure 6A,B).

**Figure 7:**
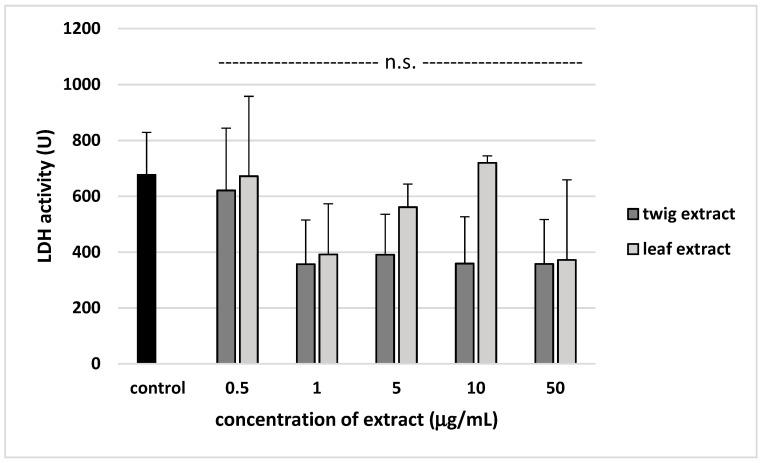
The toxic effects of twig and leaf extract (0.5–50 µg/mL; 30 min) against human blood platelets. In these experiments, blood platelets not treated with plant extract were used as control samples (positive control). Data represent mean ± SE of 6 healthy volunteers (each experiment performed in triplicate). None of the five different concentrations of the tested extract (0.5, 1, 5, 10 and 50 µg/mL) had any statistically significant effect compared to controls (*p* > 0.05 (n.s.)).

Regarding the cytotoxicity of the extracts, none were found to cause lysis of blood platelets (*p* > 0.05) (Figure 7).

In comparative experiments (for blood platelet adhesion), the extract of sea buckthorn twigs (at the tested concentration, 10 µg/mL) turned out to be more effective than 10 µg/mL aronia extract (*p* < 0.05) (Table 1). 

## 3. Discussion

In addition to multivitamin and multimineral compounds, demand is growing for supplements based on plant sources. One such plant is sea buckthorn, which offers great promise as a supplement, mainly due to its high concentrations of vitamin C, tocopherols and carotenoids, as well as its unique profile of lipids (especially unsaturated fatty acids) and various bioactive compounds, including phenolics, believed to be good for human health [8,21,22], in both berries and berry products [8,21]. 

Sea buckthorn berries are therefore good candidates for functional food production. Recently, other studies have indicated that sea buckthorn leaves and twigs are also good sources of phenolic compounds with various biological activities, including antioxidant and anticoagulant properties [17,23,24,25,26,27,28]. However, the effect of extracts from sea buckthorn leaves and twigs on blood platelet activation, which play an important role in various cardiovascular diseases, has not yet been studied. Therefore, the main aim of our in vitro study was to examine the anti-platelet properties of sea buckthorn leaf and twig extracts. 

A significant new finding is that these extracts demonstrate antiadhesive activity in the tested system of isolated washed human blood platelets, with the tested extracts reducing blood platelet adhesion to collagen and fibrinogen. In addition, both tested extracts inhibited the aggregation of platelets following thrombin stimulation; interestingly, this inhibition was not observed in the platelets activated by collagen or ADP. It is possible that that tested extracts might interact with the plasma proteins present in platelet-rich plasma (PRP), thus preventing anti-aggregatory activity.

Although blood platelet function is known to involve thiol groups [29,30], our findings do not suggest that the sea buckthorn twig and leaf extracts influenced the levels of thiol groups, and that the extracts probably do not modulate platelet activation by thiol groups. In addition, the tested extracts did not change the platelet concentration of GSH: an important physiological antioxidant.

Blood platelet activation is associated with arachidonic acid metabolism, in which different intermediate products, including pro-thrombotic thromboxane A_2_ (TXA_2_) are produced. Thromboxane A_2_ is an unstable compound, which is metabolized to inactive thromboxane B_2_ after about 30 s. In the present experiments, TBARS concentration was used as an indicator of enzymatic peroxidation of arachidonic acid in the thrombin-stimulated platelets. The twig extract was found to reduce the thrombin-induced enzymatic cascade of arachidonic acid metabolism in blood platelets. It is possible that this extract may restore the level of platelet response by helping maintain the redox balance in thrombin-activated blood platelets.

It is known that ROS, which may behave as secondary signaling molecules, are generated both in resting platelets and those activated by various agonists, including thrombin. For example, O_2_^−^ generation is associated with the enzymatic pathway of arachidonic acid metabolism. A reduction in O_2_^−^ production was observed in thrombin-activated blood platelets treated with the twig extract, which was accompanied by a decrease in TBARS production. This inhibition of TBARS production and O_2_^−^ generation by the twig extract suggests that it may also inhibit the thrombin-activated arachidonic acid pathway. It is also possible that the tested leaves and twig extracts may influence platelet reactivity by modifying other signal pathways, not only ROS level, e.g., through the inhibition of enzymatic peroxidation of lipids (TXA_2_ biosynthesis), or modifying the expression of platelet receptors.

The differences in blood platelet activation displayed by the extracts may be accounted for by differences in their phenolic profiles. For example, the greater potency of the twig extract may be associated with its higher proanthocyanidin concentration compared to the leaf extract; for example, it demonstrated greater ability to encourage the inhibiting of platelet adhesion (stimulated by thrombin) to type I collagen. Type I is the most prevalent form of collagen in the arterial vessels changed by atherosclerosis. 

Two important aspects of the use of natural compounds as drugs or supplements are their toxicity and bioavailability; these parameters are often determined for phenolics intended for use as ingredients of supplements or drugs in in vitro and in vivo models. Our present results demonstrate that none of the tested sea buckthorn extracts induced damage to human blood platelets within the whole tested concentration range. As the concentrations of sea buckthorn extracts used in the study may be achievable in blood during their oral supplementation [31,32,33], we can confirm that sea buckthorn leaves and twigs are safe for use in supplements. Gupta et al. [34] also demonstrated no cytotoxicity and side effects for sea buckthorn leaves following oral administration. 

A novel finding of our present study is that sea buckthorn twig extract (at the used concentration, 10 µg/mL), similar to well-known aronia berry extract, has anti-platelet potential. It is also an interesting that sea buckthorn twig extract had stronger anti-adhesive activity than aronia berry extract. 

The bioavailability of phenolic compounds varies according to food source. Moreover, it may also depend on the presence of various other compounds in the food matrix, including those with anti-platelet properties [35]. Tormanovic et al. [36] indicate that hippuric acid, a phenolic compound, acts as a key metabolite following consumption of fruits, including berries; this exerts anti-platelet activity by blocking the ADP receptors present on blood platelets [37].

Chong et al. [38] indicate that anthocyanidins and procyanidins have a beneficial impact on pathologies of the cardiovascular system, including platelet hyperactivity. Reis et al. [39] also report that anthocyanins have a beneficial cardiovascular effect in animal and human studies. In addition, phenolic extracts from berries and commercial products made from berries (for example from aronia berries, Aronox^®^, which has a high concentration of anthocyanidins) have been found to be more effective anti-platelet factors than pure phenolic compounds in both in vitro and in vivo models [6,38,40,41]. These findings may suggest that phenolic compounds have synergistic inhibitory actions. Our present findings regarding the extract of sea buckthorn twigs, which is rich in proanthocyanidins, are consistent with those of these studies. Therefore, we suppose that this extract may also demonstrate anti-platelet potential in an in vivo model. The action of the tested ellagitannin-rich extract from sea buckthorn leaves on blood platelet activation may depend on the interaction of the ellagitannins with thrombin and other proteins. Dong et al. [42] report that these compounds may inhibit the catalytic activity of thrombin. However, no information exists on the effect of urolithins, metabolites produced in the gut following consumption of ellagitannins, upon blood platelet function.

Our present findings shed new light on the anti-platelet potential of sea buckthorn twig and leaf extracts, particularly those of the twig extract. In future, both may be recommended in the prevention and treatment of cardiovascular diseases associated with hyperactivation of platelets. In addition, our findings may assist the development of further potential anti-platelet supplements or potent drugs against cardiovascular diseases as alternatives to classical drugs such as aspirin, which often induce side-effects. It is important that fruits are harvested together with leaves and twigs; these by products represent a rich source of additional safe phenolic compounds with anti-platelet potential, which would otherwise be regarded as production waste.

## 4. Materials and Methods

### 4.1. Chemicals

ADP was obtained from Chrono-Log Corporation (Havertown, USA). Thrombin was purchased from BioMed Lublin, Poland. Collagen type I, bovine serum albumin (BSA), cytochrome C, 5,5′-dithio-bis(2-nitro-benzoic acid), and dimethylsulfoxide (DMSO) were purchased from Sigma (St. Louis, MO, USA). Fibrinogen was isolated from pooled citrated human plasma by cold ethanol precipitation followed by ammonium sulphate fractionation at 26% saturation at 4 ˚C, according to Doolittle [43]; its concentration was determined spectrophotometrically at 280 nm using an extinction coefficient of 1.55 for 1 mg/mL solution. The concentration of purified human fibrinogen in the reaction system was 2 mg/mL. All other reagents represented analytical grade and were provided by commercial suppliers.

The content of phenolics in the phenolic-rich powder aronia berr extract (commercial product: Aronox^®^ by Agropharm Ltd., Poland; batch No. 020/2007k) amounted to 309.6 mg/g of extract, including phenolic acids (isomers of chlorogenic acid), 149.2 mg/g of extract, anthocyanins (anthocyanin glycosides: cyanidin 3-galactoside, cyanidin 3-glucoside, cyanidin 3-arabinoside, cyanidin 3-xyloside), 110.7 mg/g, and flavonoids (quercetin glycosides), 49.7 mg/g of extract. The high-performance liquid chromatography (HPLC) determination of this extract was described previously [18,19,20].

### 4.2. Plant Material

Sea buckthorn twigs and leaves were obtained from a horticultural farm in Sokółka, Podlaskie Voivodeship, Poland (53°24′N, 23°30′E), the largest Polish producer of sea buckthorn fruits. The plant material was identified by Mr. Stanislaw Trzonkowski, the owner of the farm. Voucher specimens have been deposited at the Institute of Soil Science and Plant Cultivation, Sate Research Institute, Pulawy, Poland (IUNG/HRH/2015/2).

### 4.3. Chemical Characteristics of Extracts from Sea Buckthorn Twigs and Leaves

The extracts from leaves and twigs of sea buckthorn were prepared as previously described (Sadowska et al., 2017). Briefly, freeze-dried leaves and air-dried twigs were milled in a laboratory mill (Retsch ZM200, Germany). The powdered plant material (140 g of the leaves or 200 g of the twigs) was extracted with 3 L (in three batches) of 80% methanol (*v/v*), for 48 h, at room temperature; the extraction was assisted by ultrasonic treatment (6 × 10 min.). After filtration, the methanol extracts were defatted with n-hexane. The defatted extract was concentrated under reduced pressure, the residue was resuspended in Milli-Q water, acidified with formic acid and subjected to n-butanol extraction. The butanol extracts obtained were rotary evaporated, and the residue was suspended in Milli-Q water and freeze-dried. Their composition was determined by reverse-phase ultra high-performance liquid chromatography–mass spectrometry (UHPLC–MS), using the ACQUITY UPLC^TM^ system (Waters, Milford, MA, USA), coupled with and ACQUITY TQD (Waters) triple quadrupole mass detector. Samples were chromatographed using an ACQUITY BEH C18 (100 mm × 2.1 mm, 1.7 μm; Waters) column. MS analyses were performed using negative and positive ion mode. More details of the applied analytical method are presented elsewhere [26]. Constituents of the extracts were classified and identified on the basis of their ultraviolet (UV) and MS spectra (including in-source fragmentation), authentic standards, as well as literature data [44,45,46,47]. UV-DAD detection (range: from 190 to 480 nm) was used for semi-quantitation of phenolic compounds. The content of individual hydrolysable tannins, flavonoids and proanthocyanidins was expressed as gallic acid, rutin and epicatechin equivalents, respectively, and was determined on the basis of calibration curves. The peak integration of hydrolysable tannins was performed at 270 nm, flavonoids were determined at 350 nm, and proanthocyanidins at 280 nm. Shown results are means ± SD of three replications.

The principal constituents of sea buckthorn leaf extract were ellagitannins (259.6 ± 3.1 mg/g). The total flavonoid content was 74.7 ± 0.7 mg/g. Catechin and proanthocyanidins were also detected, their total content was 7.2 ± 0.2 mg/g The sea buckthorn twig extract consisted mainly of B–type proanthocyanidins and catechin (total content 597.1 ± 10.2 mg/g). Ellagic acid and its glycosides were also present (the total content 22.4 ± 0.11 mg/g). Flavonoids were present in trace amounts (the total content was 1.7 ± 0.4 mg/g). Major phenolic constituents of the sea buckthorn leaf extract and the twig extract are shown in Table 2 and Table 3, respectively. More details on the composition can be found elsewhere [26].

Stock solutions of the twig extract and leaf extract were made in 50% DMSO. The final concentration of DMSO in samples was lower than 0.05% and its effect was determined in all experiments. A stock solution of aronia berry extract was made in H_2_O.

### 4.4. Blood Platelet Isolation

Fresh human blood was obtained from 9 healthy volunteers (non-smokers and non-drugs, including supplements with anti-platelet and antioxidative properties; median age = 27) in the Lodz Medical Center (Lodz, Poland); the samples were collected in CPD solution (citrate/phosphate/dextrose; 9:1; *v*/*v* blood/CPD). The samples were not pooled. To obtain platelet-rich plasma, whole blood was centrifuged (1200 rpm, 15 min, 25 °C). The platelet titer was determined spectrophotometrically using a Helios α spectrophotometer at a wavelength λ = 800 nm [48]. The suspension obtained was diluted with Barber buffer (0.14 M NaCl, 0.014 M Tris, 5 mM glucose, pH 7.4) to final concentration of 2 × 10^8^ cells/mL. Analysis of the blood samples was performed under the guidelines of the Helsinki Declaration for Human Research, and approved by the Committee on the Ethics of Research in Human Experimentation of the University of Lodz (resolution No. 3/KBBN-UŁ/II/2016). The first, participants provided verbal consent to the researchers, and later participants provided written the documents.

The platelet suspension was incubated for 30 min at 37 °C with extracts from individual parts of sea buckthorn at final concentrations of 0.5, 1.0, 5.0, 10 and 50 μg/mL. Moreover, blood platelets were incubated for 30 min at 37 °C with aronia extract at the final concentration of 10 μg/mL.

### 4.5. Platelet Adhesion

Platelet adhesion was evaluated by measuring the activity of the platelet exoenzyme (acid phosphatase). Platelets were dissolved with Triton X-100. The formation of *p*-nitrophenol was measured at λ = 405 nm using a SPECTROstar Nano Microplate Reader (BMG LABTECH, Germany) following the addition of *p*-nitrophenylphosphate, the phosphatase substrate. The color reaction is created by the addition of 2 M NaOH. The absorbance of control blood platelets (without tested extracts) was expressed as 100% [16,49].

### 4.6. Platelet Aggregation

Platelet aggregation was measured by turbidimetry in platelet-rich plasma or in a platelet suspended in Barber’s buffer using the optical Chrono-Log aggregometer (Chrono-Log, Havertown, PA, USA) [50].

Samples were prepared with 594 μL platelet-rich plasma (PRP) plus 6 μL extracts, or platelets suspended in Barber buffer and 6 μL tested extracts. In addition, a control sample without extract was prepared. Such prepared samples (with extract and without extract) were incubated at 37 °C for 30 min.

After incubation, 5 μL ADP (final concentrations 10 μM) or 5 μL collagen (final concentration–2 μg/mL) was added to the platelet rich plasma (PRP) for 10 min. The aggregometer was calibrated against the poor platelet plasma (100% aggregation).

After incubation, 5 μL thrombin (final concentration 1 Unit/mL) was added for 10 min to the platelets suspended in Barber’s buffer. The aggregometer was calibrated against Barber’s buffer (100% aggregation).

In these experiments, the blood platelets or PRP not treated with plant extract were used as control samples (positive control), and the rate of agonist-induced aggregation for the control sample (in the absence of plant extract) was 100%.

### 4.7. Glutathione and Thiol Group Measurement

The concentrations of thiol groups in platelet proteins and the glutathione concentration was measured spectrophotometrically using a SPECTROstar Nano Microplate Reader (BMG LABTECH, Germany) at λ = 412 nm with Ellman’s reagent: 5,5′-dithio-bis-(2-nitrobenzoic acid. The thionitrobenzoate protein derivative and the yellow thionitrobenzoic anion were formed as a result of the reaction of Ellaman’s digestion with ionised thiol groups. The concentration of thiol groups was calculated using a molar absorption coefficient (ε = 13,600 M^−1^cm^−1^) [51,52].

### 4.8. Lipid Peroxidation Measurement

Lipid peroxidation was determined by measuring TBARS concentration. First, 15% trichloroacetic acid and 0.37% thiobarbituric acid were added to the test samples. The samples were heated in a heating block at 100 °C for 10 min. After cooling, the test samples were centrifuged (10,000 rpm, 15 min, 18 °C). The absorbance of the supernatant was measured at λ = 535 nm using a SPECTROstar Nano Microplate Reader (BMG LABTECH, Germany) [17,18,53,54].

### 4.9. Superoxide Anion Measurement

The superoxide anion level in blood platelets was determined by spectrophotometric measurement of the reduction of ferricytochrome c to ferrocytochrome. Cytochrome c (160 μM) was added to two platelet suspensions: one stimulated by thrombin and another that was unstimulated (resting). The samples were centrifuged (2000× *g*). The absorbance of the supernatant was measured at λ = 550 nm using a SPECTROstar Nano Microplate Reader (BMG LABTECH, Germany). The O_2_^−^ determination uses the molar absorption coefficient for cytochrome C, which is 18,700 M^−1^cm^−1^ [22,55].

### 4.10. Lactate Dehydrogenase (LDH) Activity Measurement

Determination of the activity of lactate dehydrogenase (LDH) released from platelets is a measure of the toxicity of the tested extracts against platelets. The test samples were centrifuged (15 min, 25 °C, 2500 rpm). The microtiter plate was loaded with 270 μL of 0.1 M phosphate buffer, 10 μL of supernatant and 10 μL of NADH. After a 20-min incubation at room temperature, 10 μL of pyruvate (5 mg) was added and the absorbance was measured. The reading was repeated for 10 min in every minute using a SPECTROstar Nano Microplate Reader (BMG LABTECH, Germany) at λ = 340 nm [56].

### 4.11. Data Analysis

Several tests were used to perform the statistical analysis. In order to eliminate uncertain data, the Q-Dixon test was performed. All the values in this study were expressed as mean ± SE; n–number of blood donors. Statistical analysis was performed with one-way analysis of variance (ANOVA) for repeated measurements.

## Figures and Tables

**Table 1:** Comparison of the effects of sea buckthorn twig and leaf extracts and aronia berry extract at the concentration (10 µg/mL) on blood platelet adhesion. Results are given as means ± SE of 5–9 healthy volunteers (experiments done in triplicate).and expressed in percentage (%) difference between the control and tested extracts.

	Inhibition of Resting Blood Platelet Adhesion to Collagen (%)	Inhibition of Thrombin-Activated Platelets to Collagen (%)	Inhibition of Thrombin- Activated Platelets to Fibrinogen (%)	Inhibition of ADP-Activated Platelets to Fibrinogen (%)
Sea buckthorn twig extract (a)	39.6 ± 13.4 (*p* > 0.05, a vs. b, c)	59.4 ± 9.0 (*p* < 0.05, a vs. b, c)	63.4 ± 5.3 (*p* < 0.05, a vs. b, c)	29.0 ± 16.4 (*p* > 0.05, a vs. b, c)
Sea buckthorn leaf extract (b)	46.3 ± 15.6 (*p* > 0.05, b vs. c)	42.1 ± 10.3 (*p* > 0.05, b vs. c)	55.8 ± 9.2 (*p* < 0.05, b vs. c)	53.0 ± 29.1 (*p* > 0.05, b vs. c)
Aronia berry extract (c)	24.5 ± 11.4	34.9 ± 12.9	32.1 ± 17.4	30.7 ± 15.9

**Table 2:** Major phenolic compounds (above 5 µg/mg) of the sea buckthorn leaf extract.

No.	Compounds (Tentative Identification)	t_R_ (min)	λ max (nm)	[M − H]^−^	Fragment Ions (+) (*m*/*z*)	Concentration (µg/mg)
				[M + H]^+^		
1	strictinin/isomer	13.92	220, 270	633	153, 277, 303, 447	6.6 ± 0.3 ^a^
				635		
2	strictinin/isomer	14.12	220, 270	633	153, 277, 303, 447	7.4 ± 0.3 ^a^
				635		
3	stachyurin/isomer	14.61	222, 270	935	153, 277, 345, 617	14.2 ± 0.5 ^a^
				937		
4	casuarinin/isomer	15.13	227, 270	935	153, 255, 345, 617	24.5 ± 0.5 ^a^
				937		
5	casuarinin/isomer	15.34	230, 270	935	153, 255, 345, 617	39.4 ± 0.5 ^a^
				937		
6	hippophaenin B/isomer	17.17	224, 270	1103	153, 345, 471, 617	8.8 ± 0.2 ^a^
				1105		
7	hippophaenin B/isomer	17.30	224, 270	1103	153, 345, 471, 617	19.6 ± 1.0 ^a^
				1105		
8	ellagic acid-Hex	19.30	253, 261	463	153, 303	7.4 ± 0.2 ^a^
				465		
9	casuarictin/isomer	21.56	224, 270sh	935	153, 277, 303, 447, 785	21.7 ± 0.2 ^a^
				937		
10	Q-Hex-dHex	23.31	255, 352	609	303, 449	5.9 ± 0.0 ^b^
				611		
11	ellagic acid-Pen	24.02	253, 361	433	303	8.0 ± 0.2 ^a^
				435		
12	ellagic acid	24.37	253, 366	301		14.0 ± 0.3 ^a^
				303		
13	I-3-*O*-Hex-7-*O*-dHex	26.31	255, 350	623	317, 463	8.4 ± 0.0 ^b^
				625		
14	I-3-*O*-Glc-7-*O*-Rha	27.33	255, 352	623	317, 463	15.9 ± 0.1 ^b^
				625		
15	K-Hex-pCouA	43.85	266, 314	593	147, 287	5.4 ± 0.1 ^b^
				595		

Hex—hexose; dHex—deoxyhexose; Glc—glucose; I—isorhamnetin; K—kaempferol; pCouA—*p*-coumaric acid; Q—quercetin; Pen—pentose; Rha—rhamnose; ^a^ gallic acid equivalent; ^b^ rutin equivalent. The presented data is an updated version of a table published in the supplementary materials of the article by Sadowska et al. (2017).

**Table 3:** Major phenolic compounds (above 5 µg/mg) of the butanol extract of sea buckthorn twigs.

No.	Compounds (Tentative Identification)	t_R_ (min)	λ max (nm)	[M − H]^−^	Fragment Ions (+) (*m/z*)	Concentration (µg/mg)
				[M + H]^+^		
1	gallocatechin-catechin	5.38	270, 300sh	593	259, 305, 465	9.2 ± 0.4 ^b^
				595		
2	gallocatechin-catechin	5.85	270, 300sh	593	259, 305, 465	8.0 ± 0.4 ^b^
				595		
3	dimeric proanthocyanidin	9.71	200, 279	577	289	58.4 ± 0.7 ^b^
				579		
4	catechin	10.86	200, 278	289	139	76.1 ± 0.8 ^b^
				291		
5	trimeric proanthocyanidin	11.37	200, 279	865	139, 289, 579	23.3 ± 0.6 ^b^
				867		
6	tetrameric proanthocyanidin	14.90	200, 278	1153	289, 577, 865	12.3 ± 0.2 ^b^
				1155		
7	trimeric proanthocyanidin	16.14	200, 278	865	289, 577	13.8 ± 0.3 ^b^
				867		
8	dimeric proanthocyanidin	16.77	200, 278	577	291	7.4 ± 0.2 ^b^
				579		
9	tetrameric proanthocyanidin	19.23	200, 279	1153	289, 577, 865	11.8 ± 0.2 ^b^
				1155		
10	ellagic acid	24.46	253, 366	301		7.5 ± 0.1 ^a^
				303		

^a^ gallic acid equivalent; ^b^ epicatechin equivalent; The presented data is an updated version of a table published in the supplementary materials of the article by Sadowska et al. (2017).

## Abbreviations

ADP—adenosine diphosphate; BSA—bovine serum albumin; CPD—citrate/phosphate/dextrose; DMSO—dimethylsulfoxide; GSH—glutathione; LDH—lactate dehydrogenase; O_2_^−^—superoxide anion; PRP—platelet-rich plasma; ROS—reactive oxygen species; TBARS—thiobarbituric acid reactive substances; TXA_2_—thromboxane A_2_.
